# Genetic transformation in conifers: current status and future prospects

**DOI:** 10.48130/forres-0024-0007

**Published:** 2024-03-21

**Authors:** Huanhuan Zhao, Jinfeng Zhang, Jian Zhao, Shihui Niu

**Affiliations:** State Key Laboratory of Tree Genetics and Breeding, State Key Laboratory of Efficient Production of Forest Resources, National Engineering Research Center of Tree Breeding and Ecological Restoration, College of Biological Sciences and Biotechnology, Beijing Forestry University, Beijing 100083, China

**Keywords:** Conifer, Genetic transformation, Regeneration, Prospects

## Abstract

Genetic transformation has been a cornerstone in plant molecular biology research and molecular design breeding, facilitating innovative approaches for the genetic improvement of trees with long breeding cycles. Despite the profound ecological and economic significance of conifers in global forestry, the application of genetic transformation in this group has been fraught with challenges. Nevertheless, genetic transformation has achieved notable advances in certain conifer species, while these advances are confined to specific genotypes, they offer valuable insights for technological breakthroughs in other species. This review offers an in-depth examination of the progress achieved in the genetic transformation of conifers. This discussion encompasses various factors, including expression vector construction, gene-delivery methods, and regeneration systems. Additionally, the hurdles encountered in the pursuit of a universal model for conifer transformation are discussed, along with the proposal of potential strategies for future developments. This comprehensive overview seeks to stimulate further research and innovation in this crucial field of forest biotechnology.

## Introduction

Globally, conifers are pivotal sources of timber and pulpwood, thus holding immense economic and environmental value. The huge genome, high heterozygosity, prolonged vegetative growth period, and restricted genetic transformation system of conifers^[1−5]^ limit the availability of genetic tools for investigating their developmental regulation, resulting in sluggish research progress. Studies identifying gene function in conifers have relied on heterologous expression in angiosperm model species. Since the initial report of transgenic *Populus* in 1987^[6]^, significant strides have been made in achieving stable genetic transformation in various forest tree species. Subsequent to this, various genetic transformation systems for conifers have been reported. In 1991, *Agrobacterium rhizogenes* was employed to infect aseptic seedlings of European larch (*Larix decidua* Mill.), yielding transgenic plants with stable foreign gene expression^[7]^. Numerous *Agrobacterium* strains, leading to tumor development in a variety of coniferous species, have been identified^[7, 8]^. However, reports of successful regeneration in conifers stably transformed using *Agrobacterium*^[9−13]^, as well as stable transformation *via* particle bombardment^[14−17]^, are scarce, primarily due to inadequate regeneration procedures^[18]^. Recent developments and explorations in transgenic methods have made the mere transfer of DNA into plant cells no longer a limiting factor. Yet, the ability to regenerate complex tissues or organs after DNA transfer remains a major challenge^[19]^. Additionally, the establishment of genetic transformation systems is ongoing for most coniferous species, with successful transformation limited to a few species, often hindered by issues like low efficiency^[20]^. Currently, the focus of conifer genetic transformation is on enhancing growth rates, wood properties, pest resistance, stress tolerance, and herbicide resistance^[21−27]^.

This review offers a comprehensive overview of recent advancements in genetic transformation technologies and their applications in conifers. Influencing factors in genetic transformation encompass vector construction (*Agrobacterium* strain type, promoter types, and target genes), DNA delivery methods (*Agrobacterium*-mediated, biobombardment, and protoplast transformation), and plant regeneration pathways. We also propose various strategies to advance genetic transformation in conifers, including optimizing transformation protocols, elucidating molecular mechanisms, enhancing tissue culture techniques, overcoming cell wall barriers, exploring genetic variation, employing nanoparticle and non-tissue culture-mediated transformation, utilizing genome editing tools, and encouraging international collaboration.

## Expression vector elements

### *Agrobacterium* strains

The strains of *Agrobacterium* utilized in plant genetic transformation are categorized into three types: octopine, nopaline, and agropine (succinamopine), represented by strains LBA4404, GV3101, and EHA101/EHA105, respectively. *Agrobacterium* strains exhibit differential abilities to transform recipient material (Table 1). Humara et al. documented the transfer and expression of foreign chimeric genes in the cotyledons of *Pinus pinea*^[28]^. It was observed that EHA105, containing the plasmid p35SGUSint, demonstrated greater infectivity compared to LBA4404 or C58GV3850, with 49.7% of cotyledons exhibiting diffuse blue staining 7 d post-infection. Similarly, Le et al. employed three strains, EHA105, LBA4404, and GV3101, to facilitate the transformation of white spruce, yet only EHA105 proved effective^[29]^. In another study testing various *A. tumefaciens* strains (EHA105, GV3101, and LBA4404), the highest frequency (60%) of transient *β*-glucuronidase expression in Slash pine embryos was observed with *Agrobacterium* strain GV3101, yielding over 330 blue spots per embryo^[30]^. Liu successfully developed a high-efficiency *Agrobacterium*-mediated transient gene expression system for *P. tabuliformis* callus using strain GV3101, achieving a peak transient transformation efficiency of 70.1%^[31]^. Even within the same *Agrobacterium* strain, the effects vary significantly owing to differences in the structures of the constructed vectors. Grant et al. introduced six distinct plasmids – pMP2482, pTGUS, p4CL, pSLJ1111, pLN27, and pLUG – into *A. tumefaciens* strain KYRT1 and demonstrated that the pSLJ1111 and p4CL plasmids were markedly more effective than the others^[32]^. Consequently, trials targeting specific conifer species are essential to ascertain suitable strains for transformation.

**Table 1:** Plant expression vector construction.

Tree species	Plasmids	Strains	Genes	Promoters	Ref.
*Pinus*					
*Pinus pinea*	p35SGUSint	EHA105/LBA4404/ C58GV3850	*uidA*	*35S*	[28]
*Pinus strobus*	pGIN/pBIV/pBIVSAR/pBINm-gfp5-ER	C58pMP90	*GUS*	*35S*/2 × *35S*	[9]
	pCAMBIA1301	GV3101	*GUS*	*35S*	[10]
*Pinus taeda*	pAD1289/pToK47/pBISN1/pWWS006	LBA4404/GV3101/EHA105	*GUS*	*35S*	[51]
	pPCV6NFHygGUSINT	GV3101	*GUS*	*35S*	[52]
	pGUS3/pSSLa.3	EHA101/EHA105	*GUS*	*35S/RbcS*	[53]
	pCAMBIA1301	EHA105	*GUS*	*35S*	[54]
	pCAMBIA1301	GV3101/EHA105/LBA4404	*GUS*	*35S*	[55]
	pBIGM	LBA4404	*Mt1D*/*GutD*	*35S*	[22]
*Pinus radiata*	pBI121	LBA4404	*GUS*	*35S*	[56]
	pGA643	AGL1	*GUS*	*35S*	[11]
	pGUL/pKEA	EHA105	*NPTII*/*uidA*/*Bar*	*35S*	[57]
	pMP2482/pTGUS/ p4CL/pSLJ1111/pLN27/pLUG	KYRT1	*GFP*	*35S*/*CoA ligase 1*	[32]
*Pinus pinaster*	pPCV6NFGUS	C58pMP90	*GUS*	*35S*	[58]
	pBINUbiGUSint	EHA105/AGL1/LBA4404	*GUS*	*ubi1*	[59]
*Pinus patula*	pAHC25	LBA4404	*GUS*	*ubiquitin*	[12]
*Pinus elliottii*	pCAMBIA1301	EHA105/GV3101/LBA4404	*GUS*	*35S*	[30]
*Pinus massoniana*	pBI121	EHA105	*CslA2*	*35S*	[13]
*Pinus tabuliformis*	pBI121	GV3101	*GUS*	*35S*	[31]
*Larix*					
*Larix decidua*	pRi11325	Rhizogenes strains 11325	Ri plasmid	/	[7]
	pCGN1133/pWB139	strains 11325	*Bt*/*aroA*	*35S*	[21]
hybrid larch	pMRKE70Km	C58pMP90	*NPTII*	*35S*	[60]
	pCAMBIA1301	GV3101	*GUS*	*35S*	[61]
*Larix olgensis*	pCAMBIA1300/pBI121	GV3101	*GUS*	*35S/PtHCA2-1*	[35]
	VB191103	GV3101	*LoHDZ2*	*35S*	[25]
*Larix kaempferi*	Super1300-GFP	GV3101	*LaCDKB1;2*	*Super*	[24]
*Picea*					
*Picea sitchensis*	MOG23	LBA4404/strain 1065	*GUS*	*35S*	[62]
*Picea abies*	pAD1289/pToK47/pBISN1/pWWS006	LBA4404/GV3101/EHA105	*GUS*	*35S*	[51]
	pBIV10	C58/pMP90	*GUS*	2 × *35S*	[63]
	pET-22b	LBA4404	*Cry3A*	*35S*	[23]
*Picea mariana*	pBIV10	C58/pMP90	*GUS*	2 × *35S*	[63]
*Picea glauca*	pBIV10	C58/pMP90	*GUS*	2 × *35S*	[63]
	pBI121	EHA105/GV3101/ LBA4404	*GUS*	*35S*	[29]
	pUC19	C58pMP90	*WUS/CHAP3A*	*G10*	[64]
*Abies*					
*Abies* spp.	pTS2	AGLO	*GUS*	2 × *35S*	[65]
*Abies koreana*	pBIV10/MP90	C58/pMP90/LBA4404	*GUS*	2 × *35S*	[66]
*Taxus*					
*Taxus brevifolia/Taxus baccata*	/	Bo542/C58	/	/	[8]
*Chamaecyparis*					
*Chamaecyparis obtusa*	pBin19-sgfp	C58/pMP90	*GFP*	*35S*	[67]
*Cryptomeria*					
*Cryptomeria japonica*	pIG121-Hm/pUbiP-GFP-Hyg	GV3101/pMP90	*GFP/GUS*	*35S/ubiquitin*	[68]
	pIG121-Hm	GV3101/pMP90	*GFP*	*35S*	[69]

### Types of promoters

Although a variety of promoters are utilized in angiosperms for the genetic engineering of both monocots and dicots, their use in gymnosperms remains limited (Table 1). The cauliflower mosaic virus (*CaMV*) *35S* promoter, a prominent constitutive driver of transgene expression, is predominantly utilized in dicots^[33]^. However, despite their frequent use for gene overexpression, the activity of constitutive *CaM35S* promoters is notably lower in conifers^[34, 35]^. Constructs containing the *uidA* gene, which encodes *β*-glucuronidase (*GUS*), or the green fluorescent protein (*GFP*) gene, were introduced into embryogenic tissues to monitor the activities of these protein products over time. Expression levels of the *uidA* gene were minimal with a *35S-gus* intron construct, yet increased twentyfold when using a *35S-35S-AMVgus::nptII* construct^[9]^.

Furthermore, although the *CaM35S* promoter is functional in certain conifers, there remains a lack of efficient promoters capable of high-level, constitutive gene expression that can accommodate multiple transgenes within a single vector. Consequently, there is a need for diverse and robust promoters specifically tailored for gymnosperms, potentially in synergy with CRISPR/Cas-mediated gene editing technology^[36]^. *CmYLCV*^[37]^, isolated from *Cestrum* yellow leaf curling virus—a double-stranded DNA plant pararetrovirus of the Caulimoviridae family—demonstrates heritable, strong, and constitutive activity in both monocot and dicot species. *ZmUbi*^[38]^, a ubiquitin promoter derived from maize, exhibits high efficiency exclusively in monocot species, including maize^[38]^, wheat^[39]^, sugarcane^[40]^, rice^[41, 42]^, sorghum^[43]^, and others^[44]^. Utilizing transient expression technology in Chinese fir protoplasts, an *in vivo* molecular biological investigation compared the activities of *Cula11* and *Cula08*—constitutive expression promoters from Chinese fir—with *CaM35S*^[45, 46]^, *CmYLCV*, and *ZmUbi*, commonly used in plant genetic engineering, revealing that *Cula11* and *Cula08* exhibited higher activity^[36]^. Seven constitutive promoters underwent screening *via* a dual luciferase (LUC) transient expression assay, revealing that *PcUbi* exhibited the highest activity in *Cryptomeria japonica* embryogenic tissue and was thus deemed the most suitable promoter for driving *SpCas9* expression^[47]^. The *pCAMBIA1300-PtHCA2-1* promoter-*GUS* binary expression vector, harboring the open reading frame (ORF) of the *GUS* gene under the control of the poplar high cambial *PtHCA2–1* promoter, was subjected to testing, resulting in the observation of tissue-specific expression of the *GUS* gene in somatic embryos of transgenic larch^[35]^.

### Transformed exogenous genes

Despite significant progress in transgenic methodologies for conifers, the preponderance of exogenous genes employed thus far are screening marker genes (e.g., *uidA*, *npt II*, *hpt*, *GFP*, and *GUS*). Reports of transformations involving target genes that hold genuine potential for practical applications in production are scarce (Table 1). The initial report on the regeneration of transgenic conifer plants, specifically larch, expressing value-added genes involved herbicide and insect resistance genes *via* *Agrobacterium*-mediated gene transfer^[21]^. Some research groups have successfully transferred insect and herbicide resistance genes into various conifer species^[14−16, 23, 26, 48, 49]^. Overexpression of the *LoHDZ2* gene in the embryonic tissues of *L. olgensis* has been suggested to confer enhanced stress resistance^[25]^. Simultaneously express two genes: mannitol-1-phosphate dehydrogenase (*Mt1D*) and glucitol-6-phosphate dehydrogenase (*GutD*) enhanced tolerance to salt stress in transgenic loblolly pine^[22]^. The overexpression of the *LaCDKB1;2* gene in the embryonic tissues of *L. kaempferi* has been shown to promote cell proliferation and high-quality cotyledon embryo formation during somatic embryogenesis. This provides a foundation for examining the regulatory mechanisms of somatic embryogenesis in larch and for developing new breeding materials^[24]^. Overexpression of *WUSCHEL-related HOMEOBOX 2* (*WOX2*) during proliferation and maturation of somatic embryos of *P. pinaster* led to alterations in the quantity and quality of cotyledonary embryos^[50]^. However, reports of transformation involving target genes that possess genuine potential for practical applications remain limited.

## Gene-delivery methods in conifers

### *Agrobacterium*-mediated transformation

**Figure 1:**
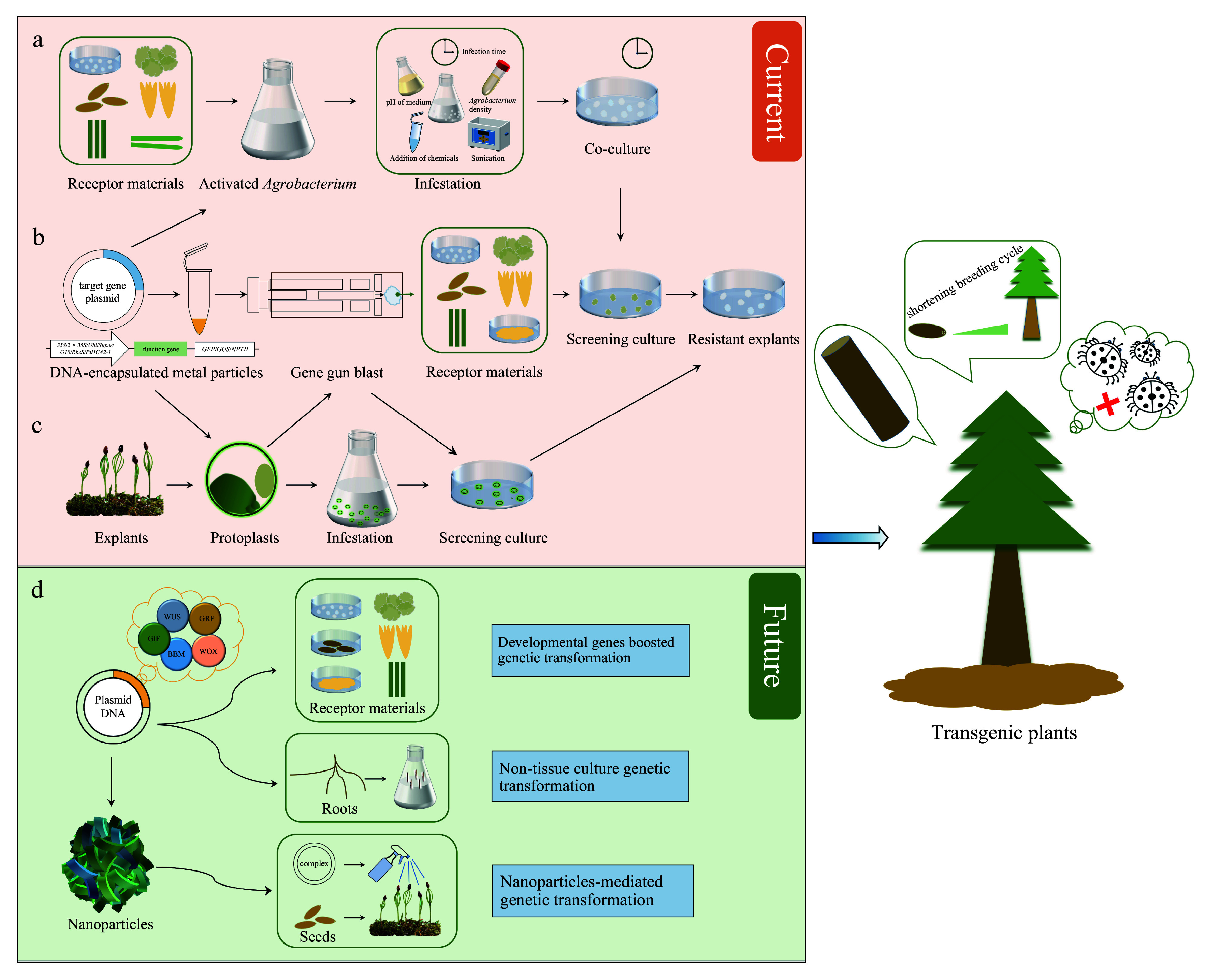
Techniques and prospects for genetic transformation of conifers. (a) *Agrobacterium*-mediated genetic transformation. (b) Genetic transformation *via* biolistic bombardment. (c) Protoplast transformation. (d) Potential strategies for transformation improvement in conifers.

*Agrobacterium*-mediated transformation represents the most prevalent method for achieving stable genetic transformation. Cell lines generated through this method demonstrate enhanced stability in transgene expression among progeny and reduced instances of transcriptional and posttranscriptional gene silencing^[19]^. However, this method encompasses several drawbacks, such as bacterial overgrowth and tissue necrosis, arising from adverse co-cultivation conditions, potentially affecting the transformation frequency^[19]^. Nevertheless, from the standpoint of conversion efficiency, it remains a valuable technology^[68]^. Since the inaugural report of conifer transformation^[7]^, there have been significant advancements in *Agrobacterium*-mediated genetic transformation. In recent years, there has made encouraging progress in the field of genetic transformation of conifers (Fig. 1a & Table 2), resulting in transgenic plants derived from European larch^[21]^, hybrid larch^[60, 61]^, white spruce^[29, 63, 64]^, Norway spruce^[23, 51]^, loblolly pine^[20, 52, 53, 55]^, and radiata pine^[11, 32, 56, 57]^.

**Table 2:** *Agrobacterium*-mediated transformation in conifers.

Tree species	Acceptor materials	Co-culture time	OD_600nm _	Results	Ref.
*Pinus*					
*Pinus pinea*	Cotyledons	3 d	1	Cotyledons forming buds	[28]
*Pinus strobus*	Embryogenic tissues	2 d	0.6	Regenerated plant	[9]
	Mature zygotic embryos	12 h	0.8−1.0	Regenerated plant	[10]
*Pinus taeda*	Embryogenic tissues	2 d	1	Transient expression	[51]
	Mature zygotic embryos	3−5 d	/	Regenerated plant	[52]
	Shoot apex	7 d	/	Transgenic plants	[53]
	Mature zygotic embryos	3−5 d	0.8−1.0	Transgenic plants	[54]
	Mature zygotic embryos	3−5 d	0.8−1.0	Transgenic plants	[55]
	Mature zygotic embryos	3−5 d	0.5−1.0	Improve salt tolerance	[22]
*Pinus radiata*	Embryogenic tissues	1 d	0.6	Stable transformation	[56]
	Cotyledons	5−60 min	OD_550nm_ = 0.4	Transgenic plants	[11]
	Embryogenic tissues	5 d	OD_550nm_ = 0.5−0.8	Transgenic plants	[57]
	Micropropagated shoot	3 d	OD_550nm_ = 0.35−0.4	Transgenic plants	[32]
*Pinus pinaster*	Embryogenic tissues	36 h	0.6	Transgenic plants	[58]
	Embryogenic tissues	3 d	0.3	Transgenic plants	[59]
*Pinus patula*	Embryogenic tissues	2 d	0.5−0.75	Transgenic tissues	[12]
*Pinus elliottii*	Mature zygotic embryos	3 d	0.9	Transgenic plants	[30]
*Pinus massoniana*	Mature zygotic embryos	3 d	0.5	Transgenic plants	[13]
*Pinus tabuliformis*	Callus/hypocotyls/Needles	3 d	0.8	Transient expression	[31]
*Larix*					
*Larix decidua*	Hypocotyls	2−3 d	/	Regenerated plant	[7]
	Hypocotyls	4 d	/	Regenerated plant	[21]
hybrid larch	Embryogenic tissues	2 d	0.3	Regenerated plant	[60]
	Embryogenic tissues	2 d	0.5	Regenerated plant	[61]
*Larix olgensis*	Embryogenic tissues	3 d	0.6	Transgenic plants	[35]
	Embryogenic tissues	2 d	0.5	Enhance stress resistance	[25]
*Larix kaempferi*	Embryogenic tissues	2 d	0.1	Promotes cell proliferation	[24]
*Picea*					
*Picea sitchensis*	Embryogenic cell lines	3 d	0.8−1.1	Stable transformation	[62]
*Picea abies*	Embryogenic tissues	2 d	1	Transient expression	[51]
	Embryogenic tissues	2 d	0.6	Transgenic plants	[63]
	Embryogenic tissues	2 d	/	Transgenic plants	[23]
*Picea mariana*	Embryogenic tissues	2 d	0.6	Transgenic plants	[63]
*Picea glauca*	Embryogenic tissues	2 d	0.6	Transgenic plants	[63]
	Embryogenic tissues	2 d	1	Transgenic plants	[29]
	Embryogenic tissues	/	/	Transgenic plants	[64]
*Abies*					
*Abies* spp.	Embryogenic tissues	2 d	0.6	Transgenic plants	[65]
*Abies koreana*	Embryogenic tissues	3 d	0.6	Transgenic plants	[66]
*Taxus*					
*Taxus brevifolia/Taxus baccata*	Shoot segments	3 d	/	Gall formation	[8]
*Chamaecyparis*					
*Chamaecyparis obtusa*	Embryogenic tissues	2 d	0.3	Transgenic plants	[67]
*Cryptomeria*					
*Cryptomeria japonica*	Embryogenic tissues	2 d	0.15	Enhance transformation	[68]
	Embryogenic tissues	2 d	0.2−0.6	Transgenic plants	[69]

Although *Agrobacterium*-mediated gene transfer is extensively employed in numerous biotechnology laboratories, its large-scale application in conifer transformation is hindered by challenges in propagating explant material, selection inefficiencies, and low transformation rates^[51]^. Wenck et al. explored co-cultivation conditions and various disarmed *Agrobacterium* strains to enhance transformation efficiency. They discovered that incorporating additional virulence genes, such as a constitutively active *virG* or extra copies of *virG* and *virB* from pTiBo542, amplified the transformation efficiency of Norway spruce by 1000-fold relative to initial experiments, which exhibited minimal or nonexistent transient expression^[51]^. Tang examined the influence of additional virulence (*vir*) genes in *A. tumefaciens* and the impact of sonication on the transformation efficiency of loblolly pine^[54]^. Utilizing plasmids with supplementary *vir* genes and sonication significantly enhanced the transfer efficiency, affecting not only transient expression but also the recovery of hygromycin-resistant lines. In their studies on *Agrobacterium*-mediated hybrid larch transformation, Levee et al. observed one to two transformation events per 100 cocultured masses^[60]^. Introducing 100 µM of coniferyl alcohol led to an increase in yield. Other studies demonstrated that sonication^[10, 30]^ and the addition of chemicals, including okadaic acid, trifluoperazine, acetosyringone, thidiazuron, and others^[10, 30, 35, 66, 70]^, significantly enhanced the transformation efficiency of conifers and further advanced the transformation system. Additionally, several groups have illustrated that cold treatment of *Agrobacterium* can augment transformation efficiency^[13]^.

Transformation frequencies depend on species, genotype, and post-cultivation protocol. In a study involving three species, *Picea mariana* was transformed at the highest frequency, followed by *P. glauca* and *P. abies*^[63]^. Furthermore, for all the species, transgenic plants were regenerated using modified protocols for somatic embryo maturation and germination. Le et al. devised an efficient method for the reproducible transformation of embryogenic white spruce tissue using *A. tumefaciens*-mediated gene transfer^[29]^. A shoot-based, genotype-independent transformation method employing *A. tumefaciens* facilitated plant recovery and enabled the transformation of elite germplasm^[53]^. Shoots from 4- to 6-week-old seedlings and adventitious shoots from cultures were inoculated with *A. tumefaciens*, underwent selection, and were subsequently regenerated. Micropropagated shoot explants from *P. radiate* have successfully been employed to produce stable transgenic plants *via* *A. tumefaciens*-mediated transformation^[32]^. It is crucial during the transformation process to inhibit and prevent contamination caused by excessive *Agrobacterium* growth. In the *A. tumefaciens*-mediated transformation of *P. pinea* cotyledons, a high cotyledon mortality rate occurs, possibly related to the plant's hypersensitive response to bacterial infection^[28]^. For conifers, non-toxic antibiotics to plant cells, like cefotaxime sodium (Cef) or timentin, are frequently incorporated into the medium. Also, in the post-transformation selection medium, selecting transformants is crucial for obtaining transgenic plants. If tissues are initially cultivated for 10 d on a medium with timentin (400 mg·L^–1^) to avert bacterial overgrowth, the recovery of kanamycin-resistant tissues is enhanced before applying selection pressure^[29]^. An evaluation of three antibiotics was conducted to assess their effectiveness in eliminating *A. tumefaciens* during the genetic transformation of loblolly pine using mature zygotic embryos^[55]^. Exposing the cultures to 350 mg·L^–1^ of carbenicillin, Cef, and timentin for a duration of up to 6 weeks failed to eliminate *Agrobacterium*; however, increasing the concentration to 500 mg·L^–1^ successfully eradicated the bacterium from co-cultured zygotic embryos^[55]^.

Identifying the optimal combination of infection time and concentration is crucial for successful conifer transgenesis during genetic transformation experiments. Generally, the bacterial solution concentration for infecting conifers is maintained at an OD_600_ of 0.3–0.8. Elevating the *Agrobacterium* concentration and extending the infection duration can result in excessive bacterial proliferation and hypersensitive necrosis of explants, thereby diminishing transformation efficiency^[28]^. Conversely, employing a low-density *Agrobacterium* suspension and a brief infection period often results in weak infectivity, which similarly reduces transformation efficiency^[13]^. Moreover, the infection duration influences T-DNA transfer and, consequently, the efficiency of genetic transformation. The infection duration, typically less than 30 min, varies depending on the explant type and the physiological status of the conifer species. However, both the concentration and infection duration of the bacterial solution must be tailored to the condition, type, and environmental factors of the explants, necessitating further research.

### Genetic transformation *via* biolistic bombardment

Particle bombardment, also known as biolistics, serves as an alternative method for plant genetic transformation, circumventing the limitations associated with *Agrobacterium*-mediated genetic transformation^[71]^. This method is not limited by biological constraints and is applicable to a broad spectrum of plant species. However, in the context of conifer transformation frequency, biolistic techniques are generally regarded as less effective than *Agrobacterium*-mediated genetic transformation^[68]^. Foreign genes have successfully been expressed in all tested conifer tissues *via* particle bombardment, encompassing embryos, seedlings, xylem, pollen, needles, buds, cell suspension cultures, embryogenic callus, cell aggregate cultures, and roots (Fig. 1b & Table 3). While most of these attempts yielded only transient expression, they have offered insightful information about the factors influencing gene expression in various tissues capable of regeneration^[20]^. *GFP* introduction into conifer tissues has been achieved through microprojectile bombardment, with transient expression subsequently observed^[72]^. The *CaMV35S* promoter facilitated *GUS* gene expression in loblolly pine tissues^[73]^. Microprojectile bombardment proves to be an effective technique for assaying transient gene expression in pine, and it harbors potential for generating transgenic pine plants. Using high-velocity microprojectiles, plasmid DNA with the *GUS* gene, under the control of the *CaMV35S* promoter, has been introduced into cultured Douglas fir cotyledons^[74]^. Additionally, the particle gun technique has been employed to transform a variety of receptor materials in different tree species, including callus and pollen of larch^[75, 76]^, Chir pine^[16]^, and Norway spruce^[14, 77−80]^. Particle bombardment has been applied to Lodgepole pine, yellow cypress, western hemlock, jack pine, and black spruce pollen to achieve transient *GUS* gene expression, demonstrating the method's viability for pollen transformation^[81]^. Furthermore, particle bombardment has facilitated the testing of transient expression of heterologous promoters in organized tissues and angiosperm promoters in gymnosperms^[82]^. Comparative analyses have been conducted on the initiation strengths of transient expression for eight distinct promoter sequences, based on the relative levels of *GUS* expression^[76]^.

**Table 3:** Biolistic bombardment genetic transformation in conifers.

Tree species	Acceptor materials	Plasmids	Promoters	Genes	Results	Ref.
*Pinus*						
*Pinus taeda*	Cotyledons	pBI221	*35S*	*GUS*	Transient expression	[73]
*Pinus radiata*	Suspension cells	pBI221	*35S*	*GUS*	Transient expression	[87]
	Embryogenic tissues	pCW103/pCWI22	2 × *35S*	*gus*A	Transient expression	[88]
	Cotyledons	pBI121/pCGUΔl/ pAIGusN/pActl-D	*35S/UbBI/Adhl/Actl*	*gus*A	Transient expression	[89]
	Embryogenic tissues	pRC101/pCW122	*35S/Emu*	*uidA*	Transgenic plants	[83]
	Embryogenic tissues	pAHC25/pCW122	*maize ubiquitin/35S*	*GUS*/*Bar*	Transgenic plants	[14]
	Calli	pCW122/pCADsense	*35S*	*npt II*/*Cad*	Transgenic calli	[90]
	Embryogenic tissues	pMYC3425/pAW16/ pCW132/pRN2	*Emu/ubi*	*Cry*1Ac	Transgenic plants	[15]
*Pinus concorta/Pinus banksiana*	Mature pollen	pBM113Kp/pRT99GUS/ pAct1-D/pGA984	*35S/rice actin*	*GUS*	Transient expression	[81]
*Pinus sylvestris*	Calli/Vegetative buds/ Suspension cells	pBI221	*35S*	*GUS*	Transient expression	[91]
	Pollen	pBI221/pRT99/pBI410/ pBI426/pBM113	*35S/* *EmP/* *UbB1*	*GUS*	Transient expression	[79 ]
*Pinus strobus*	Embryonal masses	p35S-GFP/mGFP4	*35S*	*GFP*	Transient expression	[72]
*Pinus aristata/Pinus griffithii/Pinus monticola*	Pollen tubes	pBI221	*35S*	*GUS*	Transient expression	[92]
*Pinus patula*	Embryogenic tissues	pAHC25	*35S*	*Bar*/*GUS*	Somatic embryos	[48]
*Pinus nigra*	Embryogenic tissues	pCW122	2 × *35S*	*GUS*	Somatic embryos	[86]
*Pinus roxbughii*	Mature zygotic embryos	pAHC25	*maize ubiquitin*	*Bar/GUS*	Transgenic plants	[16]
*Picea*						
*Picea glauca*	Zygotic embryos/Seedlings/ embryogenic callus	pUC19	*35S*	*GUS*	Transient expression	[82]
	Somatic embryos	pBI426	*35S*	*GUS*	Stable transformation	[93]
	Somatic embryos	pTVBT41100	*35S*	*GUS*/*Bt*	Transgenic plants	[49]
	Embryonal masses	p35S-GFP/mGFP4	*35S*	*GFP*	Transient expression	[72]
	Embryogenic tissues	pKUB/pBI426	*maize ubiquitin/35S*	*cry*1Ab	Transgenic plants	[26]
*Picea mariana*	Embryogenic tissues	pRT99GUS/pBM113Kp	*35S*	*GUS*	Transient expression	[94]
	Embryogenic tissues	pRT99GUS/pGUSInt/ pMON9909	*35S/*Em protein of whea*t/Rbcs/NOS/* *Actin/Arabin*	*GUS*	Transient expression	[76]
	Mature pollen	pBM113Kp/pRT99GUS/ pAct1-D/pGA984	*35S/rice actin*	*GUS*	Transient expression	[81]
	Embryonal masses	pRT99GUS/pBI426	*35S*	*GUS*	Transgenic plants	[84]
	Pollen/Embryonal masses/ Somatic embryos	p35S-GFP/mGFP4	*35S*	*GFP*	Transient expression	[72]
	Mature somatic embryos	pBI221.23	*35S*	*GUS*	Transgenic plants	[17]
*Picea abies*	Somatic embryo	pRT99gus	*35S*	*GUS*	Stable transformation	[77]
	Embryogenic tissues	pRT99gus/pJIT65/ Dc8gus/pBMI13Kp	*35S/*2 × *35S/* *Act1-D/Dc8*	*GUS*	Transient expression	[80]
	Pollen	pBI221/pRT99/pBI410/ pBI426/pBM113	*35S/* *EmP/* *UbB1*	*GUS*	Transient expression	[79]
	Embryogenic tissues	pCW122	*35S*	*GUS*	Transgenic plants	[95]
	Embryogenic tissues	pAHC25	*maize ubiquitin*	*Bar*	Transgenic plants	[78]
	Embryogenic tissues	pAHC25/pCW122	*maize ubiquitin/35S*	*GUS*/*Bar*	Transgenic plants	[14]
	Embryogenic tissues	pAHC25	*maize ubiquitin*	*CCR*	Transgenic plants	[27]
*Larix*						
*Larix* × *eurolepis*	Embryogenic tissues	pRT99GUS/pGUSInt/ pMON9909	*35S/*Em protein of wheat/*Rbcs/NOS/* *Actin/Arabin*	*GUS*	Transient expression	[76]
*Larix laricina*	Embryonal masses	pBI426/pRT99gus/ pRT66gus/pRT55gus	*35S/*2 × *35S*	*GUS*	Transient expression	[75]
*Larix gmelinii*	Zygotic embryos	pUC-GHG/pBI221-HPT	*35S*	*GUS/GFP*	Transgenic plants	[34]
*Pseudotsuga*						
*Pseudotsuga menziesii*	Cotyledons	pTVBTGUS	*35S*	*GUS*	Transient expression	[74]
*Chamaecyparis*						
*Chamaecyparis nootkatensis*	Mature pollen	pBM113Kp/pRT99GUS/ pAct1-D/pGA984	*35S/rice actin*	*GUS*	Transient expression	[81]
*Tsuga*						
*Tsuga heterophylla*	Mature pollen	pBM113Kp/pRT99GUS/ pAct1-D/pGA984	*35S/rice actin*	*GUS*	Transient expression	[81]
*Abies*						
*Abies nordmanniana*	Embryogenic tissues	pCW122	*35S*	*GUS*	Transgenic plants	[85]

Particle bombardment-mediated transformation is capable of regenerating whole plants. In *P. glauca* plants, the stable expression of an exogenous gene marked the first successful creation of transgenic plants using the particle gun method^[49]^. Walter et al. used a particle gun to bombard four embryonic cell lines of *P. radiate*, resulting in over 150 transgenic plants from 20 transformation experiments^[83]^. Analyses using Southern and Northern blotting confirmed the integration of the target gene into the genome. Particle bombardment facilitated the stable genetic transformation of *P. mariana* in two target tissues: mature cotyledonary somatic embryos and suspensions from embryonal masses, employing the Biolistic PDS-1000/He device^[84]^. The expression of the *GUS* gene in needles of regenerated seedlings demonstrates the potential for sustained transgene expression in spruce^[17]^. Using biolistic transformation, stable genetic transformation has been accomplished in embryogenic cultures of *Abies nordmanniana*, leading to the regeneration of transgenic plants^[85]^. A biolistic approach has successfully achieved stable transformation in embryogenic tissues of *P. nigra* Arn., specifically cell line E104^[86]^. Given its versatility and broad applicability, particle bombardment is anticipated to continue as a primary method in genetic transformation.

Particle bombardment possesses significant potential for producing transgenic conifer plants. A key objective in tree breeding involves reducing lignin content or modifying its composition, which would aid in delignification during pulping processes. When the antisense construct of the *cinnamoyl CoA reductase* (*CCR*) gene was introduced into Norway spruce, a significant reduction in the total lignin content of dry wood was observed compared to controls^[27]^. Lachance et al. conducted a study on the accumulation of *cry*lAb protein in embryogenic tissues, somatic seedling needles, and 5-year-old field-grown needles of white spruce^[26]^. Insect feeding trials, both in the laboratory and the field, indicated that multiple transgenic spruce lines proved lethal to spruce budworm larvae. Through biolistic transformation of embryogenic tissue, transgenic radiata pine plants harboring the *Bacillus thuringiensis* (*Bt*) toxin gene, *cry*1Ac, were successfully produced^[15]^. Ongoing research is being conducted on functional genes utilizing this technology^[14, 16, 78]^.

### Protoplast transformation

Protoplast technology enables various unique approaches to the genetic improvement of plants^[96]^. Protoplast transient expression assays serve as versatile tools in genomics, transcriptomics, metabolic, and epigenetic studies^[97]^. Coupling protoplast transient expression experiments with high-resolution imaging enables simple, rapid, and efficient analysis and characterisation of gene functions and regulatory networks. This includes protein subcellular localisation, protein-protein interactions, transcriptional regulatory networks, and gene responses to external cues^[98−100]^. Reporter genes commonly used, like *LUC* and *GUS*, are employed to assess gene activity in conifer protoplasts^[87]^. *P. glauca* protoplasts were transformed with the *chloramphenicol acetyltransferase* (*CAT*) reporter gene through electroporation^[101]^. Fir and pine protoplasts were successfully transformed with the *LUC* gene through electroporation, with gene expression enhanced by the addition of polyethylene glycol (PEG) to the mixture^[102]^. Developments in methods for transient gene expression have been made for protoplasts of black spruce and jack pine^[103]^. In electroporated protoplasts of *P. glauca*, *P. mariana*, and *P. banksiana*, the activity levels of exogenous genes depend on the promoter, electroporation conditions, and the targeted cell line^[104]^. A new transient transformation system for Chinese fir protoplasts has been established, achieving cell wall regeneration and protoplast division. This method serves as a reference for conducting functional studies on Chinese fir-related genes^[105]^. However, the challenges in establishing protoplast regeneration systems in conifers mean that protoplast-based genetic transformation studies primarily focus on transient gene expression and the investigation of gene function and expression regulation (Fig. 1c).

## Receptor materials and regeneration systems

Establishing an effective and stable regeneration system is crucial for rapidly expanding conifer populations for seedling production and successful heritage transformation. A range of plant materials, each with unique advantages, serves as transformation receptors for conifers. These include zygotic embryos, hypocotyls, embryonic tissues, somatic embryos, protoplasts, stem tips, and pollen^[7, 10, 13, 31, 51, 53, 81, 101]^. Embryonic tissues have been the focus of extensive research as receptors in numerous studies^[9, 27, 35, 51, 57, 58, 85]^. Additionally, *Agrobacterium*-mediated genetic transformation using mature zygotic embryos as explants has been successfully implemented in *P. taeda*^[22, 52, 54, 55]^, *P. elliottii*^[30]^, and *P. massoniana*^[13]^. Cotyledons and hypocotyls are identified as suitable explants for genetic transformation^[7, 11, 21, 28]^. Currently, embryonic tissue of conifers is predominantly used as recipient material through the somatic embryogenesis pathway to obtain stably-transformed regenerated plants (Tables 2 &  3).

A primary challenge in the genetic transformation of coniferous trees involves plant regeneration^[106]^. This challenge arises primarily from the unique biological properties and regeneration mechanisms of conifers. Tissue culture in conifers proves more challenging than in other plants. This is attributed to the cells of conifers, especially those from mature trees, which have a lower capacity for differentiation and regeneration^[107]^. The tissue culture process entails inducing cells or tissues from the parent plant to develop into new plants under controlled conditions, a process notably less efficient in conifers. Furthermore, during tissue culture, particularly over extended periods, the genetic stability of conifers may be affected. Cell division and differentiation, occurring during tissue culture, may introduce genetic mutations; additionally, genome doubling, leading to the formation of polyploids, can also occur. Consequently, even if plant regeneration is successful, the resultant plants may exhibit genetic variations, potentially posing challenges in subsequent applications and research^[19, 106]^. The regeneration of conifer tissue is notably sensitive to the balance of plant hormones and other culture conditions. Different species of conifers often require specific combinations of hormones and culture environments, thereby complicating the identification of a universal method applicable to all types^[108]^. Conifers generally exhibit a long regeneration process, which implies that the entire process from tissue culture to mature plant consumes a considerable amount of time, acting as a limiting factor in research and application. Variations in regeneration capabilities among different species of conifers are notable.

In summary, although the genetic transformation and regeneration of coniferous trees are theoretically feasible, their practical implementation is fraught with several challenges, most notably in tissue culture efficiency, genetic stability maintenance, and adaptation to different species' characteristics^[109]^. Addressing these challenges necessitates in-depth research and substantial technological innovation.

## Challenges in conifer genetic transformation

Despite the numerous promising success cases mentioned, it must be acknowledged that genetic transformation continues to pose a significant challenge for most conifer researchers. To date, none of these methods have proven universally applicable across multiple species or varied genotypes. Consequently, while a method may appear promising, it often remains confined to successful implementation under specific laboratory conditions, lacking widespread applicability. Significant progress is still required to develop a universal model for conifers that is as straightforward, efficient, and reproducible as those established for angiosperm model species.

### Complex biology

Conifers possess distinct and complex biological characteristics, setting them apart from commonly utilized genetic engineering plants like *Arabidopsis* or tobacco. Their prolonged generation times, expansive genomes, and elaborate reproductive processes contribute to the challenges in working with them^[1, 2, 4]^.

### Low transformation efficiency

Despite the establishment of transformation protocols, the efficiency of integrating foreign genes into the conifer genome frequently remains low^[54, 63]^. Consequently, only a minor fraction of transformed cells effectively express the introduced gene, posing significant challenges in producing stable and predictable genetically modified organisms.

### Species variability

Various conifer species exhibit unique biological traits and varying responses to transformation techniques. A technique effective in one conifer species might not yield similar results in another, necessitating tailored optimization for each species.

### Genetic complexity

The size and complexity of conifer genomes pose challenges in the introduction and expression of foreign genes. A thorough understanding of the regulatory elements and mechanisms within conifer genomes is crucial for genetic engineering success^[3−5]^. However, such knowledge is typically less comprehensive than that available for model plant species.

### Tissue culture challenges

Conifers often require specialized tissue culture techniques for regeneration and propagation. Developing suitable tissue culture methods for conifers, particularly those compatible with genetic transformation, is a significant hurdle. Studies have indicated that the induction rate of embryogenic tissues from immature seeds in conifers is influenced by both the genotype and the embryonic developmental stage^[110, 111]^.

### Phenolic compounds

Conifers, like many plants, contain high levels of phenolic compounds, such as lignins and polyphenols^[112, 113]^. These compounds may exert inhibitory effects on the enzymes used in the genetic transformation process. Phenolic compounds are known to contribute to oxidative stress, DNA degradation, and may interfere with the integration of foreign genes into the plant genome.

### Secondary metabolites

Conifers produce a diverse array of secondary metabolites, including terpenoids and flavonoids, which can potentially affect the success of genetic transformation. These compounds can exhibit toxic effects on the transformed cells or may interfere with the activity of introduced genes.

### Cell wall composition

The cell walls of conifers are notably complex and rigid, serving to provide structural support to the plant. However, this complexity may impede the delivery of foreign DNA into plant cells. Efficient transformation frequently necessitates overcoming these barriers to ensure that the introduced genetic material successfully reaches the nucleus of the target cells^[114, 115]^.

### Genetic variation

The presence of genetic variation within conifer populations may influence the success of genetic transformation. Individuals within a species often exhibit varying responses to transformation protocols, and optimizing these protocols for broader applicability presents a significant challenge.

Addressing these biochemical factors typically necessitates the development of specialized techniques and treatments within the genetic transformation process. For instance, researchers might utilize tissue culture conditions designed to mitigate the effects of phenolic compounds, or employ specialized methods to enhance the delivery of foreign DNA through the cell wall.

Comprehending the biochemical makeup of conifers and customizing transformation methods to suit their unique characteristics is an active area of research. Advances in biotechnology, encompassing the development of more robust transformation protocols and the elucidation of genes involved in stress responses, may play a pivotal role in surmounting these biochemical barriers in the future.

## Further research focus and strategies

Addressing the challenges associated with the genetic transformation of conifers necessitates a comprehensive approach that integrates advancements across multiple key domains (Fig. 1d). The following delineates potential strategies and focal areas.

### Optimization of transformation protocols

It is imperative for researchers to persist in refining and optimizing transformation protocols tailored to various conifer species. This encompasses enhancing the efficiency of introducing foreign genes into conifer cells and developing uniform methods applicable across diverse species. The utilization of developmental genes may prove beneficial in promoting transformation. These genes, capable of acting through diverse developmental mechanisms to enhance the regeneration of transgenic cells, have seen extensive use in model plants to stimulate embryogenesis and, in some instances, organogenesis^[116−118]^. In summary, the overexpression of regeneration-regulating transcription factors, including BBM, WUS2, WOX5, GRF4, and GIF1, could enhance genetic transformation in conifers characterized by low regeneration efficiency, substantial transformation difficulty, and genotype limitation.

### Understanding molecular mechanisms

Gaining a deeper understanding of the molecular and biochemical processes in conifers is essential. This necessitates research into the regulation of gene expression, understanding the role of secondary metabolites, and comprehending the response of conifers to stress conditions. This knowledge is crucial in informing the development of transformation methods that are synergistic with the unique biology of conifers.

### Tissue culture advances

The improvement of tissue culture techniques, crucial for supporting the regeneration and propagation of conifer plants, is vital. The development of protocols for efficient plant regeneration from transformed cells can significantly bolster the success of genetic transformation. Conversely, most prevailing methods for plant genome modification entail regenerating plants from genetically modified cells in tissue culture, a process that is technically challenging, costly, time-consuming, and limited to a narrow range of plant species or genotypes^[119]^. Cao et al. outlined a notably straightforward cut–dip–budding (CDB) delivery system, which includes inoculating explants with *A. rhizogenes*, subsequently generating transformed roots that yield transformed buds through root suckering^[120]^. The advancement of methods that circumvent laborious procedures, such as tissue culture, and facilitate obtaining transgenic and gene-edited plants, marks a significant breakthrough in conifer research.

### Overcoming cell wall barriers

Exploring strategies to overcome the challenges presented by the complex cell walls of conifers is imperative. This could involve employing enzymes or other agents to facilitate the penetration of foreign DNA into plant cells. In the realm of conifer biotechnology, the initial protoplast extraction in *P. contorta* laid the foundation for the establishment of a transient transformation system in conifers^[121]^.

### Understanding genetic variation

Recognizing and addressing genetic variation within conifer species is critical. Customizing transformation protocols to accommodate the diverse genetic backgrounds of individuals within a species can lead to broader success in genetic transformation^[122]^.

### Application of advanced biotechnologies

The utilization of cutting-edge biotechnological tools, notably CRISPR/Cas9 gene editing, can offer more precise control over the modification of conifer genomes. These advanced technologies have the potential to overcome several challenges associated with traditional genetic transformation methods. Genome editing represents a powerful technology for functional genomic research and trait improvement. Cui et al. successfully achieved knockout of the *DXS1* gene in white spruce (*P. glauca*) employing the conifer-specific CRISPR/Cas9 toolbox^[123]^. Recently, CRISPR/Cas9-mediated targeted mutagenesis has been demonstrated in radiata pine^[124]^, Japanese cedar^[47]^, and Chinese fir^[36]^, underscoring its feasibility in conifers. This represents a potent genome editing system of significant importance for gene function studies and the genetic improvement of plant traits, likely to make substantial contributions to the development of molecular breeding in conifers.

### Nanoparticle-mediated genetic transformation

In future research endeavors, the use of nanomaterials for genetic modification promises to expand the scope of plant molecular research, particularly for conifers, which currently lack efficient systems for regeneration and stable genetic transformation. Nanocarriers are characterized by their large surface area, facilitating efficient gene loading, alongside high biocompatibility to safeguard the loaded genes, coupled with low toxicity and enhanced safety^[125, 126]^. Consequently, nanoparticles hold the potential to be utilized in developing transgenic technologies for conifer regeneration without dependency on tissue culture, potentially overcoming the technical challenges in genetic transformation of recalcitrant plant genotypes. Conversely, the exploration of stable and targeted nanocarrier-mediated gene editing technologies offers the prospect of achieving genetic improvements in conifers.

### Ecological considerations

Considering the ecological significance of conifers, comprehensive risk assessments and detailed ecological studies should accompany all attempts at genetic modification. Comprehending the potential environmental impact and addressing public concerns are imperative for the responsible and sustainable deployment of genetically modified conifers.

### International collaboration

Given the global distribution of conifers, international collaboration among researchers, institutions, and regulatory bodies is essential to foster the sharing of knowledge, resources, and expertise. Such collaborative efforts can significantly accelerate progress and enhance the effectiveness in addressing challenges.

Sustained research and ongoing technological advancements, in conjunction with a holistic and interdisciplinary approach, are crucial to unlocking the full potential of genetic transformation in conifers, while simultaneously ensuring the responsible and ethical application of these technologies.

## Conclusions

Many reports have documented the successful expression of exogenous genes in conifers using *Agrobacterium*-mediated, particle bombardment-mediated, and protoplast-based genetic transformation methods. However, the genetic transformation of conifers faces several challenges, including low transformation efficiency, high dependence on recipient genotypes, difficulties in plant regeneration. Overall, the genetic transformation of conifers remains heavily reliant on extensive experience and sophisticated technical skills, rendering its widespread application challenging for most conifer researchers. Overcoming these challenges will usher in a new era of productivity and quality in forestry. Several potential strategies have been proposed to improve conifer transformation, including the optimization of transformation protocols, understanding molecular mechanisms, improving tissue culture techniques, overcoming cell wall barriers, understanding genetic variation, employing nanoparticle- and non-tissue culture-mediated genetic transformation, utilizing genome editing tools, fostering international collaboration, and more. In conclusion, with the ongoing development of molecular biotechnology and enhancement of various regeneration and transformation systems, research on the genetic transformation of conifer species is poised for continued progress and broader applicability.

## Author contributions

The authors confirm contribution to the paper as follows: study conception and design: Zhao J, Niu S, Zhang J; draft manuscript preparation: Zhao H; Figure creation: Zhao H. All authors reviewed the results and approved the final version of the manuscript.

## Data availability

Data sharing is not applicable to this article as no datasets were generated or analyzed during the current study.

## References

[1] (2022). The Chinese pine genome and methylome unveil key features of conifer evolution. Cell.

[2] (2022). Chinese pine (*Pinus tabuliformis* Carr.). Trends in Genetics.

[3] (2015). Improved white spruce (*Picea glauca*) genome assemblies and annotation of large gene families of conifer terpenoid and phenolic defense metabolism. The Plant Journal.

[4] (2016). Sequence of the sugar pine megagenome. Genetics.

[5] (2017). Erratum to: An improved assembly of the loblolly pine mega-genome using long-read single-molecule sequencing. GigaScience.

[6] (1987). *Agrobacterium* mediated transformation and regeneration of *Populus*. Molecular & General Genetics.

[7] (1991). *Agrobacterium rhizogenes*-mediated genetic transformation and regeneration of a conifer: *Larix decidua*. In Vitro Cellular & Developmental Biology - Plant.

[8] (1994). Genetic transformation of mature *Taxus*: an approach to genetically control the in vitro production of the anticancer drug, taxol. Plant Science.

[9] (1999). Stable genetic transformation of white pine (*Pinus strobus* L.) after cocultivation of embryogenic tissues with Agrobacterium tumefaciens. Molecular breeding.

[10] (2007). Okadaic acid and trifluoperazine enhance *Agrobacterium*-mediated transformation in eastern white pine. Plant Cell Reports.

[11] (2004). Transgenic *Pinus radiata* from *Agrobacterium tumefaciens-*mediated transformation of cotyledons. Plant Cell Reports.

[12] (2008). An *Agrobacterium*-mediated system for gene transfer in *Pinus patula*. South African Journal of Botany.

[13] (2018). Study on factors influencing transformation efficiency in *Pinus massoniana* using *Agrobacterium tumefaciens*. Plant Cell, Tissue and Organ Culture (PCTOC).

[14] (2001). Conifer genetic engineering: transgenic *Pinus radiata* (D. Don) and *Picea abies* (Karst) plants are resistant to the herbicide Buster. Plant Cell Reports.

[15] (2005). Insect-resistant transgenic *Pinus radiata*. Plant Cell Reports.

[16] (2006). Stable transformation of mature zygotic embryos and regeneration of transgenic plants of chir pine (*Pinus roxbughii* Sarg.). Plant Cell Reports.

[17] (2000). Hygromycin resistance is an effective selectable marker for biolistic transformation of black spruce (*Picea mariana*). Plant Cell Reports.

[18] (2001). Conifer genetic engineering: Particle bombardment and *Agrobacterium*-mediated gene transfer and its application in future forests. Journal of Forestry Research.

[19] (2016). Genetic transformation and somaclonal variation in conifers. Plant Biotechnology Reports.

[20] (2003). Genetic transformation of conifers and its application in forest biotechnology. Plant Cell Reports.

[21] (1994). Transgenic larch expressing genes for herbicide and insect resistance. Canadian Journal of Forest Research.

[22] (2005). Enhanced tolerance to salt stress in transgenic loblolly pine simultaneously expressing two genes encoding mannitol-1-phosphate dehydrogenase and glucitol-6-phosphate dehydrogenase. Plant Physiology and Biochemistry.

[23] (2013). Norway spruce (*Picea abies*) genetic transformation with modified *Cry3A* gene of *Bacillus thuringiensis*. Acta Biochimica Polonica.

[24] (2021). Over-expression of the cell-cycle gene *LaCDKB1;2* promotes cell proliferation and the formation of normal cotyledonary embryos during *Larix kaempferi* somatic embryogenesis. Genes.

[25] (2022). Genetic transformation of *LoHDZ2* and analysis of its function to enhance stress resistance in *Larix olgensis*. Scientific Reports.

[26] (2007). Expression of a *Bacillus thuringiensis cry1Ab* gene in transgenic white spruce and its efficacy against the spruce budworm (*Choristoneura fumiferana*). Tree Genetics & Genomes.

[27] (2008). Lignin biosynthesis in transgenic Norway spruce plants harboring an antisense construct for cinnamoyl CoA reductase (CCR). Transgenic Research.

[28] (1999). *Agrobacterium tumefaciens*-mediated transformation of *Pinus pinea* L. cotyledons: an assessment of factors influencing the efficiency of *uidA* gene transfer. Plant Cell Reports.

[29] (2001). An improved procedure for production of white spruce (*Picea glauca*) transgenic plants using *Agrobacterium tumefaciens*. Journal of Experimental Botany.

[30] (2014). Slash pine genetic transformation through embryo cocultivation with *A. tumefaciens* and transgenic plant regeneration. In Vitro Cellular & Developmental Biology - Plant.

[31] (2020). An efficient system for *Agrobacterium*-mediated transient transformation in *Pinus tabuliformis*. Plant Methods.

[32] (2015). Genetic transformation of micropropagated shoots of *Pinus radiata* D. Don. bioRxiv.

[33] (1985). Identification of DNA sequences required for activity of the cauliflower mosaic virus 35S promoter. Nature.

[34] (2005). Stable genetic transformation of *Larix gmelinii* L. by particle bombardment of zygotic embryos. Plant Cell Reports.

[35] (2020). Stable and Efficient Agrobacterium-mediated genetic transformation of larch using embryogenic callus. Frontiers in Plant Science.

[36] (2023). Application of a novel strong promoter from Chinese fir (*Cunninghamia lanceolate*) in the CRISPR/Cas mediated genome editing of its protoplasts and transgenesis of rice and poplar. Frontiers in Plant Science.

[37] (2003). Cestrum yellow leaf curling virus (CmYLCV) promoter: a new strong constitutive promoter for heterologous gene expression in a wide variety of crops. Plant Molecular Biology.

[38] (1992). Maize polyubiquitin genes: structure, thermal perturbation of expression and transcript splicing, and promoter activity following transfer to protoplasts by electroporation. Plant Molecular Biology.

[39] (2005). Improvement of wheat drought and salt tolerance by expression of a stress-inducible transcription factor *GmDREB* of soybean (Glycine max). Chinese Science Bulletin.

[40] (2003). Comparative expression analysis of two sugarcane polyubiquitin promoters and flanking sequences in transgenic plants. Journal of Plant Physiology.

[41] (1993). Activity of a maize ubiquitin promoter in transgenic rice. Plant Molecular Biology.

[42] (1994). Non-systemic expression of a stress-responsive maize polyubiquitin gene (Ubi-1) in transgenic rice plants. Plant Molecular Biology.

[43] (2012). Expression pattern of the alpha-kafirin promoter coupled with a signal peptide from *Sorghum bicolor* L. Moench. Journal of Biomedicine and Biotechnology.

[44] (1996). Ubiquitin promoter-based vectors for high-level expression of selectable and/or screenable marker genes in monocotyledonous plants. Transgenic Research.

[45] (1989). Multiple *cis* regulatory elements for maximal expression of the cauliflower mosaic virus _35_S promoter in transgenic plants. The Plant Cell.

[46] (1990). Combinatorial and synergistic properties of CaMV 35S enhancer subdomains. The EMBO Journal.

[47] (2021). CRISPR/Cas9-mediated targeted mutagenesis in Japanese cedar (*Cryptomeria japonica* D. Don). Scientific Reports.

[48] (2004). A biolistic approach towards producing transgenic *Pinus patula* embryonal suspensor masses. Plant Growth Regulation.

[49] (1993). Stable transformation of *Picea glauca* by particle acceleration. Bio/Technology.

[50] (2022). Constitutive overexpression of a conifer *WOX2* homolog Affects somatic embryo development in *Pinus pinaster* and promotes somatic embryogenesis and organogenesis in *Arabidopsis* seedlings. Frontiers in Plant Science.

[51] (1999). High-efficiency *Agrobacterium*-mediated transformation of Norway spruce *(Picea abies*) and loblolly pine (*Pinus taeda*). Plant Molecular Biology.

[52] (2001). Regeneration of transgenic loblolly pine (*Pinus taeda* L.) from zygotic embryos transformed with *Agrobacterium tumefaciens*. Planta.

[53] (2002). Transformation and regeneration of loblolly pine: shoot apex inoculation with *Agrobacterium*. Molecular Breeding.

[54] (2003). Additional virulence genes and sonication enhance *Agrobacterium tumefaciens*-mediated loblolly pine transformation. Plant Cell Reports.

[55] (2004). Effects of antibiotics on the elimination of *Agrobacterium tumefaciens* from loblolly pine (*Pinus taeda*) zygotic embryo explants and on transgenic plant regeneration. Plant Cell, Tissue and Organ Culture.

[56] (2002). Stable transformation of *Pinus radiata* embryogenic tissue by *Agrobacterium tumefaciens*. Plant Cell, Tissue and Organ Culture.

[57] (2005). Consistent and stable expression of the *nptII, uidA* and *bar* genes in transgenic *Pinus radiata* after *Agrobacterium tumefaciens*-mediated transformation using nurse cultures. Plant Cell Reports.

[58] (2006). Stable *Agrobacterium*-mediated transformation of embryogenic tissues from *Pinus pinaster* Portuguese genotypes. Plant Growth Regulation.

[59] (2013). Stable *Agrobacterium* -mediated transformation of maritime pine based on kanamycin selection. The Scientific World Journal.

[60] (1997). *Agrobacterium tumefaciens*-mediated transformation of hybrid larch (*Larix kaempferi* T L. *decidua*) and transgenic plant regenerationn. Plant Cell Reports.

[61] (2021). Embryogenic callus induction from immature zygotic embryos and genetic transformation of *Larix kaempferi* 3x *Larix gmelinii* 9. PLoS ONE.

[62] (1997). Expression of the *gus A* gene in embryogenic cell lines of Sitka spruce following *Agrobacterium*-mediated transformation. Journal of Experimental Botany.

[63] (2001). Regeneration of transgenic *Picea glauca, P. mariana*, and *P. abies* after cocultivation of embryogenic tissue with *Agrobacterium tumefaciens*. In Vitro Cellular & Developmental Biology - Plant.

[64] (2010). Hormonally regulated overexpression of *Arabidopsis WUS* and conifer *LEC1* (*CHAP3A*) in transgenic white spruce: implications for somatic embryo development and somatic seedling growth. Plant Cell Reports.

[65] (2009). *Agrobacterium*-mediated transformation of embryogenic tissues of hybrid firs (*Abies* spp.) and regeneration of transgenic emblings. Biotechnology Letters.

[66] (2014). *Agrobacterium*-mediated transformation via somatic embryogenesis system in Korean fir (*Abies koreana* Wil.), a Korean native conifer. Korean Journal of Plant Resources.

[67] (2005). *Agrobacterium tumefaciens*-mediated transformation of embryogenic tissue and transgenic plant regeneration in *Chamaecyparis obtusa* Sieb. et Zucc. Plant Cell Reports.

[68] (2013). High-efficiency *Agrobacterium*-mediated transformation of *Cryptomeria japonica* D. Don by co-cultivation on filter paper wicks followed by meropenem treatment to eliminate *Agrobacterium*. Plant Biotechnology.

[69] (2020). A protocol for *Agrobacterium*-mediated transformation of Japanese cedar, Sugi (*Cryptomeria japonica* D. Don) using embryogenic tissue explants. Plant Biotechnology.

[70] (2013). Organic nitrogen composition of the tissue culture medium influences *Agrobacterium tumefaciens* growth and the recovery of transformed *Pinus radiata* embryonal masses after cocultivation. In Vitro Cellular & Developmental Biology - Plant.

[71] (2020). Particle bombardment technology and its applications in plants. Molecular Biology Reports.

[72] (1997). Expression of the green fluorescent protein gene in conifer tissues. Plant Cell Reports.

[73] (1991). Transient expression from microprojectile-mediated DNA transfer in *pinus taeda*. Plant Cell Reports.

[74] (1991). Transient gene expression of microprojectile-introduced DNA in Douglas-fir cotyledons. Plant Cell Reports.

[75] (1997). *Larix laricina* (tamarack): somatic embryogenesis and genetic transformation. Canadian Journal of Forest Research.

[76] (1992). Effect of promoter sequence on transient expression of the β-glucuronidase gene in embryogenic calli of *Larix* × *eurolepis* and *Picea mariana* following microprojection. Canadian Journal of Botany.

[77] (1992). Genetic transformation of Norway spruce (*Picea abies* (L.) Karst) using somatic embryo explants by microprojectile bombardment. Plant Molecular Biology.

[78] (2000). Basta tolerance as a selectable and screening marker for transgenic plants of Norway spruce. Plant Cell Reports.

[79] (1997). Gene transfer by particle bombardment to Norway spruce and Scots pine pollen. Canadian Journal of Forest Research.

[80] (1994). Biological factors affecting transient transformation in embryogenic suspension cultures of *Picea abies*. Journal of Plant Physiology.

[81] (1994). Transient chimeric gene expression in pollen of five conifer species following microparticle bombardment. Canadian Journal of Forest Research.

[82] (1991). Expression of inducible angiosperm promoters in a gymnosperm, *Picea glauca* (white spruce). Plant Molecular Biology.

[83] (1998). Stable transformation and regeneration of transgenic plants of *Pinus radiata* D. Don. Plant Cell Reports.

[84] (1996). Stable genetic transformation of *Picea mariana* (Black spruce) via microprojectile bombardment. In Vitro - Plant.

[85] (2005). Stable genetic transformation of embryogenic cultures of *Abies nordmanniana* (nordmann fir) and regeneration of transgenic plants. In Vitro Cellular & Developmental Biology - Plant.

[86] (2005). Stable transformation of embryogenic tissues of *Pinus nigra* Arn. using a biolistic method. Biotechnology Letters.

[87] (1992). Expression of luciferase and β-glucuronidase in *Pinus radiata* suspension cells using electroporation and particle bombardment. Canadian Journal of Forest Research.

[88] (1994). A biolistic approach for the transfer and expression of a *gusA.* reporter gene in embryogenic cultures of *Pinus radiata*. Plant Cell Reports.

[89] (1996). Factors affecting transient gene expression in cultured radiata pine cotyledons following particle bombardment. Physiologia Plantarum.

[90] (2003). Cell differentiation, secondary cell-wall formation and transformation of callus tissue of *Pinus radiata* D. Don. Planta.

[91] (1994). Transient beta-glucuronidase expression in Scots pine tissues derived from mature trees. Canadian Journal of Forest Research.

[92] (2000). Transient gene expression in pine pollen tubes following particle bombardment. Plant Cell Reports.

[93] (1993). Transformation of white spruce (*Picea glauca*) somatic embryos by microprojectile bombardment. Plant Cell Reports.

[94] (1991). Transient expression of the β-glucuronidase gene in embryogenic callus of *Picea mariana* following microprojection. Plant Cell Reports.

[95] (1999). An efficient Biolistic® transformation protocol for *Picea abie*s embryogenic tissue and regeneration of transgenic plants. Canadian Journal of Forest Research.

[96] (2005). Plant protoplasts: status and biotechnological perspectives. Biotechnology Advances.

[97] (2022). Protoplasts: small cells with big roles in plant biology. Trends in Plant Science.

[98] (2020). Efficient isolation of protoplasts from rice calli with pause points and its application in transient gene expression and genome editing assays. Plant Methods.

[99] (2021). Optimization of protoplast isolation, transformation and its application in sugarcane (*Saccharum spontaneum* L). The Crop Journal.

[100] (2022). An efficient and universal protoplast isolation protocol suitable for transient gene expression analysis and single-cell RNA sequencing. International Journal of Molecular Sciences.

[101] (1988). Transient gene expression in electroporated *Picea glauca* protoplasts. Plant Cell Reports.

[102] (1988). Somatic proembryo formation and transient expression of a luciferase gene in Douglas fir and loblolly pine protoplasts. Plant Science.

[103] (1989). Factors affecting transient gene expression in electroporated black spruce (*Picea mariana*) and jack pine (*Pinus banksiana*) protoplasts. Theoretical and Applied Genetics.

[104] (1990). The effects of promoter on transient expression in conifer cell lines. Theoretical and Applied Genetics.

[105] (2021). Establishment of high-efficiency callus induction and transient transformation system of Chinese fir. Molecular Plant Breeding.

[106] (1986). Tissue culture and the propagation and genetic improvement of conifers: problems and possibilities. Tree Physiology.

[107] (2018). In vitro propagation of conifers using mature shoots. Journal of Forestry Research.

[108] (1988). *In vitro* propagation of *Araucaria cunninghamii* and other species of the araucariaceae via axillary meristems. Australian Journal of Botany.

[109] (1986). Tissue culture in forestry: economic and genetic potential. The Forestry Chronicle.

[110] (2003). Somatic embryogenesis and plant regeneration from immature zygotic embryos of *Cryptomeria japonica* D. Don. Plant Cell Reports.

[111] (2017). Somatic embryogenesis of immature *Cunninghamia lanceolata* (Lamb.) hook zygotic embryos. Scientific Reports.

[112] (2011). Phenolic compounds that confer resistance to spruce budworm. Entomologia Experimentalis et Applicata.

[113] (2013). Antioxidant potential of bark extracts from boreal forest conifers. Antioxidants.

[114] (1996). Transient and stable electrotransformations of intact black Mexican sweet maize cells are obtained after preplasmolysis. Plant Cell Reports.

[115] (2018). Genetic transformation of cell-walled plant and algae cells: delivering DNA through the cell wall. Briefings in Functional Genomics.

[116] (2018). Opportunities for innovation in genetic transformation of forest trees. Frontiers in Plant Science.

[117] (2016). Morphogenic regulators *Baby boom* and *Wuschel* improve monocot transformation. The Plant Cell.

[118] (2017). Selectable marker independent transformation of recalcitrant maize inbred B73 and sorghum P898012 mediated by morphogenic regulators *BABY BOOM* and *WUSCHEL2*. Plant Cell Reports.

[119] (2023). Direct delivery and fast-treated *Agrobacterium* co-culture (Fast-TrACC) plant transformation methods for *Nicotiana benthamiana*. Nature Protocols.

[120] (2023). Cut–dip–budding delivery system enables genetic modifications in plants without tissue culture. The Innovation.

[121] (1983). Isolation and growth of protoplasts from cell suspensions of *Pinus contorta* Dougl. ex Loud. Plant Cell Reports.

[122] (2021). Adaptive evolution in a conifer hybrid zone is driven by a mosaic of recently introgressed and background genetic variants. Communications Biology.

[123] (2021). Efficient multi-sites genome editing and plant regeneration *via* somatic embryogenesis in *Picea glauca*. Frontiers in Plant Science.

[124] (2021). Genome editing with CRISPR/Cas9 in *Pinus radiata* (D. Don). BMC Plant Biology.

[125] (2010). Evidence of RNAi in humans from systemically administered siRNA via targeted nanoparticles. Nature.

[126] (2010). A novel intracellular protein delivery platform based on single-protein nanocapsules. Nature Nanotechnology.

